# British Hospitals Association: Scottish Regional Committee

**Published:** 1921-02-05

**Authors:** 


### British Hospitals Association : Scottish Regional Committee.

A Regional Committee in Scotland of the British Hospitals Association was formed at a meeting of hospital managers and administrators, held in the Christian Institute, Glasgow, on January 25, 1921. Colonel D. J. Mackintosh, C.B., M.V.O., one of the Vice-Presidents of the Association, occupied the chair. The Regional Committee, it was pointed out, would be an integral part of the British Hospitals Association, and only those who joined the parent body would be eligible for membership. The Committee will consider matters affecting voluntary hospitals, and take such steps to safeguard their interests as may be considered necessary. Colonel J. A. Roxburgh was appointed Chairman for the ensuing year. Dr. D. Campbell Suttie is the Hon. Secretary. An Executive Committee, composed of representatives of groups of hospitals, was appointed.

